# Supplementary material for: Lipidomics Analysis of Human HMC3 Microglial Cells in an In Vitro Model of Metabolic Syndrome
Source: Biomolecules. 2024 Sep 30;14(10):1238. doi: 10.3390/biom14101238 (PMC11506612; doi:10.3390/biom14101238)
Supplement: Supplementary file 1 [file biomolecules-14-01238-s001.zip › biomolecules-3171537-supplementary.pdf]

Supplementary Materials

Figure S1. Heatmaps of significantly altered metabolites based on lipidomics.

A heat map of the significantly altered lipids in HMC3 cells pre-stimulated for 24 h with 25 ng/mL TNFα and then treated for 24 h with LPS (10 ng/mL), PA (200 μM), or LPS+PA. NC, negative control; VC, vehicle control (BSA). The colors in the heat map indicated the log-transformed values of each metabolite.

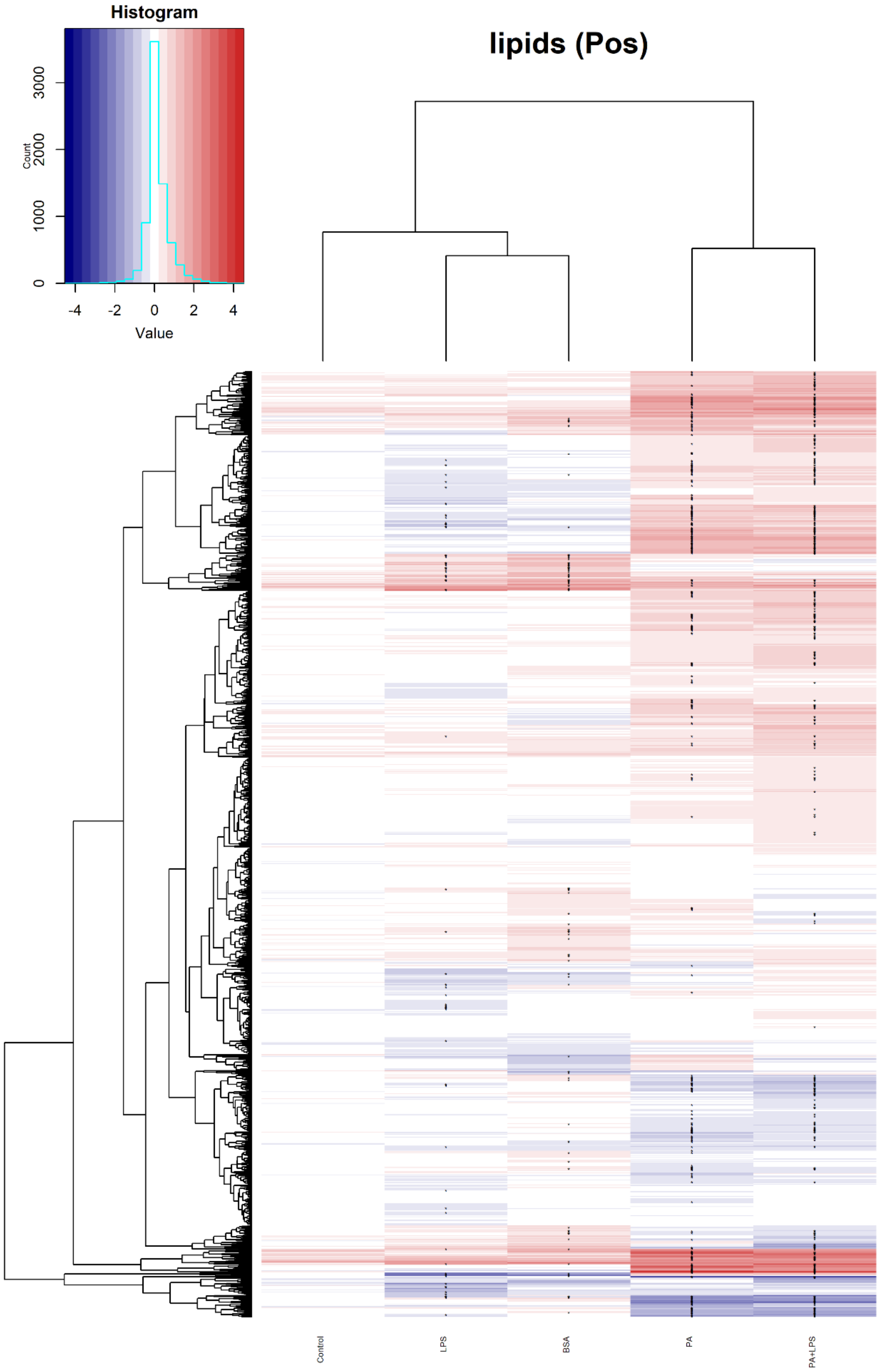

Histogram
Count
0
1000
2000
3000
-4
-2
0
2
4
Value
lipids (Pos)
Control
LPS
BSA
PA
PA+LPS

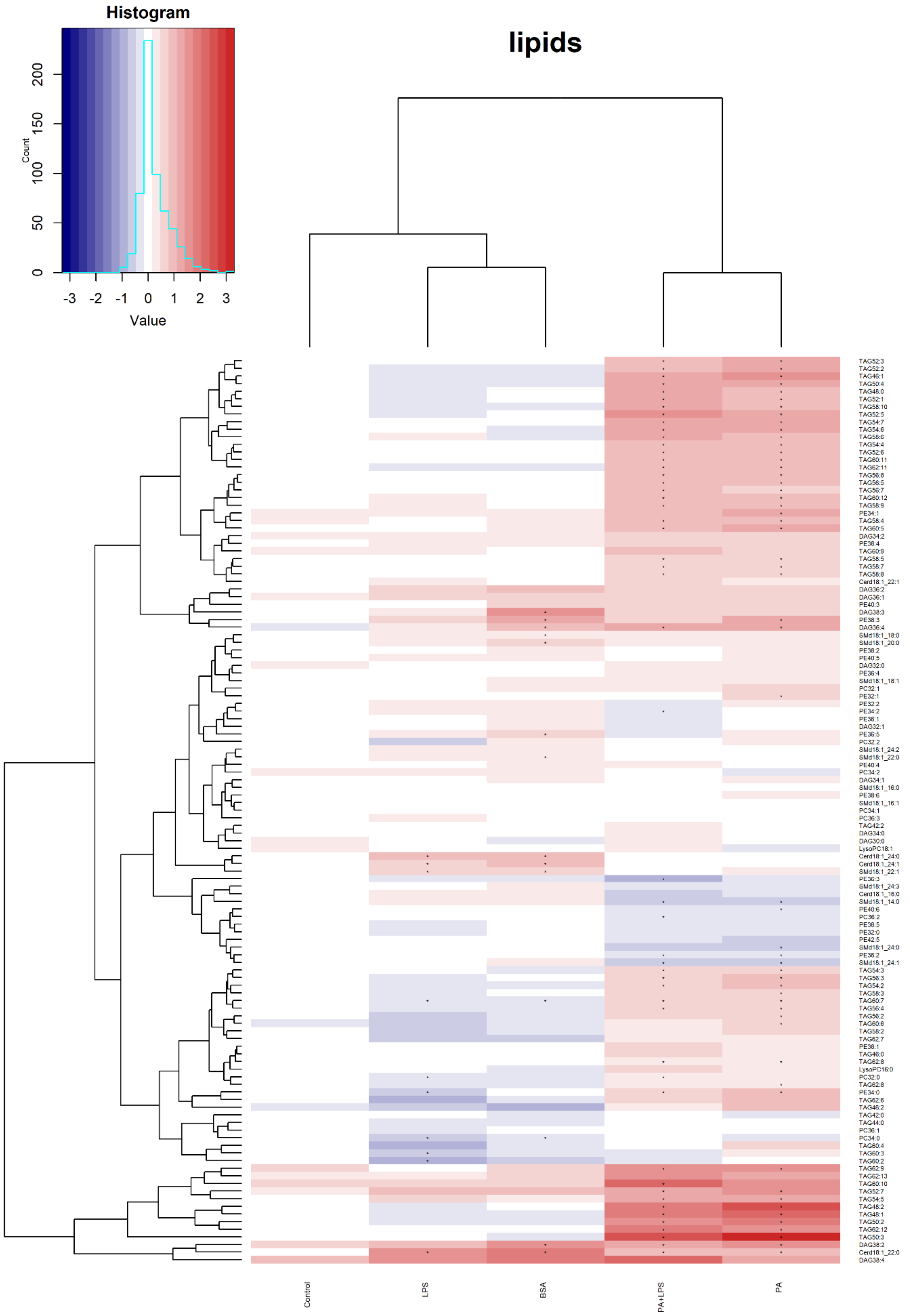

Histogram
Count
0
50
100
150
200
-3
-2
-1
0
1
2
3
Value
lipids
TAG52:3
TAG52:2
TAG46:1
TAG50:4
TAG48:0
TAG52:1
TAG58:10
TAG52:5
TAG54:7
TAG54:6
TAG58:6
TAG54:4
TAG52:6
TAG60:11
TAG62:11
TAG56:8
TAG56:5
TAG56:7
TAG60:12
TAG58:9
PE34:1
TAG58:4
TAG60:5
DAG34:2
PE38:4
TAG60:9
TAG58:5
TAG58:7
TAG58:8
Cerd18:1_22:1
DAG36:2
DAG36:1
PE40:3
DAG38:3
PE38:3
DAG36:4
SMd18:1_18:0
SMd18:1_20:0
PE38:2
PE40:5
DAG32:0
PE36:4
SMd18:1_18:1
PC32:1
PE32:1
PE32:2
PE34:2
PE36:1
DAG32:1
PE36:5
PC32:2
SMd18:1_24:2
SMd18:1_22:0
PE40:4
PC34:2
DAG34:1
SMd18:1_16:0
PE38:6
SMd18:1_16:1
PC34:1
PC36:3
TAG42:2
DAG34:0
DAG30:0
LysoPC18:1
Cerd18:1_24:0
Cerd18:1_24:1
SMd18:1_22:1
PE36:3
SMd18:1_24:3
Cerd18:1_16:0
SMd18:1_14:0
PE40:6
PC36:2
PE38:5
PE32:0
PE42:5
SMd18:1_24:0
PE36:2
SMd18:1_24:1
TAG54:3
TAG56:3
TAG54:2
TAG58:3
TAG60:7
TAG56:4
TAG56:2
TAG60:6
TAG58:2
TAG62:7
PE38:1
TAG46:0
TAG62:8
LysoPC16:0
PC32:0
TAG62:8
PE34:0
TAG62:6
TAG46:2
TAG42:0
TAG44:0
PC36:1
PC34:0
TAG60:4
TAG60:3
TAG60:2
TAG62:9
TAG62:13
TAG60:10
TAG52:7
TAG54:5
TAG48:2
TAG48:1
TAG50:2
TAG62:12
TAG50:3
DAG38:2
Cerd18:1_22:0
DAG38:4
Control
LPS
BSA
PA+LPS
PA
